# Anesthetic management of pheochromocytoma and paraganglioma for patients with Fontan circulation: a case series

**DOI:** 10.1186/s40981-023-00605-z

**Published:** 2023-03-10

**Authors:** Yuto Tanaka, Makoto Sumie, Takuma Hashimoto, Sayaka Osawa, Yuji Karashima, Tadashi Kandabashi, Ken Yamaura

**Affiliations:** 1 Department of Anesthesiology and Critical Care Medicine, Kyushu University Hospital, 3-1-1 Maidashi, Higashi-Ku, Fukuoka, 812-8582 Japan; 2 Department of Anesthesia, Saiseikai Fukuoka General Hospital, 1-3-46 Tenjin, Chuo-Ku, Fukuoka, 810-0001 Japan; 3 Department of Anesthesia, St. Mary’s Hospital, 422 Tsubukuhonmachi, Kurume, Fukuoka 830-8543 Japan; 4 Department of Critical Care, Kyushu University Hospital, 3-1-1 Maidashi, Higashi-Ku, Fukuoka, 812-8582 Japan; 5 Operating Rooms, Kyushu University Hospital, 3-1-1 Maidashi, Higashi-Ku, Fukuoka, 812-8582 Japan; 6 Medical Information Center, Kyushu University Hospital, 3-1-1 Maidashi, Higashi-Ku, Fukuoka, 812-8582 Japan

**Keywords:** Fontan circulation, Pheochromocytoma, Paraganglioma, Vasopressin, Retroperitoneal laparoscopic

## Abstract

**Background:**

Anesthetic management of pheochromocytoma and paraganglioma with Fontan circulation is challenging for physicians, with attention to cardiovascular physiology.

**Case presentation:**

We performed anesthetic management for pheochromocytoma and paraganglioma in three patients with Fontan circulation. We maintained intraoperative central venous pressure at preoperative level under fluid infusion and administrating nitric oxide to decrease pulmonary arterial resistance. We administered noradrenaline or vasopressin if low blood pressure was present despite adequate central venous pressure. Although noradrenaline is prevalent for the case of noradrenaline-secreting tumor especially after resection, we could maintain blood pressure to administrate vasopressin without increasing central venous pressure. Retroperitoneal laparoscopic approach which could avoid intra-abdominal adhesions might be selectable as case 3.

**Conclusions:**

Sophisticated management is required for pheochromocytoma and paraganglioma with Fontan circulation.

**Supplementary Information:**

The online version contains supplementary material available at 10.1186/s40981-023-00605-z.

## Background

Fontan circulation is an iatrogenic definitive palliative condition for patients with single ventricle physiology. The patient with Fontan circulation can be sensitive and vulnerable to change systemic vascular resistance (SVR), pulmonary vascular resistance (PVR), and inotropy. Pheochromocytoma and paraganglioma (P-PGL) are neuroendocrine tumors with catecholamine hypersecretion. The number of patients with Fontan circulation and P-PGL is expected to increase because survival rate of the patients is improved and P-PGL have a tendency to develop under cyanotic heart disease [1, 2]. Chronic hypoxia exposure followed by aberrant activation of common hypoxia cellular pathways and hypoxia-inducible factors in chromaffin cells appear to mechanism for the pathogenesis of P-PGL [1]. Although several case reports of P-PGL with Fontan circulation have been reported, the information on its perioperative anesthetic management is sparse (online only Supplement) [3–13].

## Case presentation

Patients’ profiles and preoperative data are summarized in Table 1, and intraoperative anesthetic profiles in Table 2.

**Table 1:** Preoperative characteristics of the three cases

Characteristics	Case 1	Case 2	Case 3
Age (years)	30	35	35
Sex	Male	Male	Female
Height (cm)	167	167	140
Weight (kg)	51	58	37
Tumor type	Paraganglioma	Paraganglioma	Pheochromocytoma paraganglioma
Surgery	Open surgery	Open surgery	Retroperitoneal laparoscopic surgery
SpO2 (%)	97 (room air)	99 (room air)	88 (O_2_ 2 l/min)
SVEF (%)	63	52	70
CVP (mmHg)	13	14	20
PVR (wood units)	0.7	1.1	NA
CI (l/min/m^2^)	3.6	2.2	1.8
PT-INR	1.94	1.82	1.64
APTT (s)	43	41	41
Antiplatelet drug	-	-	Aspirin
Anticoagulant drug	Warfarin	Apixaban	Warfarin
Heparin bridging	+	-	+

*APTT* Activated partial thromboplastin time, *CI* Cardiac index, *CVP* Central venous pressure, *INR* International normalized ratio of partial thrombin time, *NA* Not available, *SVEF* Systemic ventricular ejection fraction, *PVR* Pulmonary vascular resistance, *SpO2* Oxygen saturation

**Table 2:** Intraoperative profiles of the three cases

Profiles	Case 1	Case 2	Case 3
Anesthetic methods	General anesthesia, rectus sheath block, IVPCA	General anesthesia, transversus abdominis plane block, IVPCA	General anesthesia, local anesthesia, IVPCA
Anesthetic drugs	Sevoflurane, remifentanil	Sevoflurane, remifentanil	Sevoflurane, remifentanil
Monitoring	Standard monitoring, ABP, CVP	Standard monitoring, ABP, CVP	Standard monitoring, ABP, CVP
Nitric oxide	+	+	+
Inotropes	Milrinone, noradrenaline	Milrinone, vasopressin	Dobutamine, noradrenaline, vasopressin
Intraoperative CVP (mmHg)	10–14	12–18	12–25
Blood transfusion (ml)	560 (RCC) 480 (FFP)	-	280 (RCC)
Operation duration (min)	373	335	183
Amount of bleeding (g)	720	150	110
Extubation location	Operating room	Operating room	ICU on the first postoperative day

*ABP* Arterial blood pressure, *CVP* Central venous pressure, *FFP* Fresh-frozen plasma, *ICU* Intensive care unit, *IVPCA* Intravenous patient-controlled analgesia, *RCC* Red cell concentrate

### Case 1

The patient underwent Fontan surgery at 12 years of age for double outlet right ventricle (DORV), atrioventricular septal defect (AVSD), and pulmonary stenosis (PS). He had a history of a right adrenalectomy for pheochromocytoma at 19 years of age. The 24-h urine collection for catecholamines revealed elevated normetanephrine levels of 1.07 [normal range (NR) 0.09–0.33] µg/day and elevated noradrenaline levels of 418.2 (*NR* 48.6–168.4) µg/day. ^123^I-metaiodobenzylguanidine (MIBG) scintigraphy showed a mass accumulation around the right renal vein at the level of the second lumbar spine and the right side of the abdominal aorta at the level of the third lumbar spine. He was diagnosed with paraganglioma as a local recurrence of pheochromocytoma and scheduled for open tumor resection. He started oral administration of the α_1_-adrenergic receptor blocker doxazosin at 1.5 mg/day. His vitals were as follows: blood pressure (BP), 121/81 mmHg; heart rate (HR), 82 beats/min in sinus rhythm; and oxygen saturation (SpO_2_) in room air, 97%.

Induction of general anesthesia with fentanyl 8 µg/kg, midazolam 0.1 mg/kg, and rocuronium 1 mg/kg was successful. Anesthesia was maintained with sevoflurane 0.6–0.8 minimum alveolar concentration (MAC) and remifentanil 0.15–0.4 µg/kg/min. A continuous infusion of nitroglycerin 0.5 µg/kg/min, milrinone 0.25 µg/kg/min, and inhalation of nitric oxide (NO) 10 ppm were administered. We maintained CVP at 10–14 mmHg. We adjusted pressure control ventilation (PCV) to set peak pressure at 14 cmH_2_O and positive end expiratory pressure (PEEP) at 5 cmH_2_O. The partial pressure of arterial carbon dioxide (PaCO_2_) was maintained at 34–37 mmHg. Despite hemodynamic fluctuations during tumor manipulation (approximately ± 30% in mean arterial pressure and HR compared to before manipulation), the patient was successfully managed with three bolus infusions of nicardipine 0.2 mg and three bolus infusions of landiolol 2.5 mg. We administered a continuous infusion of noradrenaline 0.02–0.05 µg/kg/min to maintain BP after tumor resection. The operation was performed as scheduled.

### Case 2

The patient underwent Fontan surgery at 12 years of age due to DORV, AVSD, and PS. He had a medical history of right adrenalectomy for pheochromocytoma at 19 and 30 years of age. The 24-h urine collection for catecholamines revealed elevated normetanephrine levels of 1.21 µg/day and noradrenaline levels of 470.3 µg/day. ^123^I-MIBG scintigraphy showed mass accumulations around the para-aortic area. He was diagnosed with a local recurrence of paraganglioma and scheduled for open tumor resection. His vitals were as follows: BP, 131/98 mmHg; HR, 85 beats/min; and SpO_2_ in room air, 99%.

Induction of general anesthesia with fentanyl 8 µg/kg, midazolam 0.05 mg/kg, and rocuronium 0.9 mg/kg was successful. Anesthesia was maintained with sevoflurane 0.6–0.8 MAC and remifentanil 0.1–0.2 µg/kg/min. We administered a continuous infusion of milrinone 0.25 µg/kg/min, vasopressin 0.1–1 units/h, and inhalation of NO 10 ppm. We maintained CVP at approximately 15 mmHg. We adjusted PCV with PEEP to maintain a PaCO_2_ of 35–40 mmHg. Although hemodynamic fluctuations occurred during the tumor manipulation (approximately ± 20% of the mean BP compared to before manipulation), the patient was successfully managed with two bolus infusions of phenylephrine 0.1 mg and three bolus infusions of nicardipine 0.2 mg. The operation was performed as scheduled.

### Case 3

The patient had undergone Fontan surgery at 9 years of age for hypoplastic left heart syndrome and PS. She had a medical history of open tumor resection for paraganglioma at 16 years of age. Although total cavopulmonary connection conversion had been planned, local recurrence of P-PGL was accidentally revealed during the preoperative examination. The 24-h urine collection for catecholamines revealed elevated normetanephrine levels of 0.56 µg/day, noradrenaline levels of 236.6 µg/day, and dopamine levels of 1727.1 (*NR* 365.0–961.5) µg/day. ^123^I-MIBG scintigraphy showed mass accumulations around the right adrenal gland and para-aortic area. Her vitals were as follows: BP, 90/53 mmHg; HR, 65 beats/min; and SpO_2_ on O_2_ 2 l/min, 88%. Multidisciplinary preoperative discussions were conducted among the medical team consisting of anesthesiologists, cardiologists, surgeons, and endocrinologists. Our team could have consensus as follows. Retroperitoneal laparoscopic approach might be better than intra-abdominal laparoscopic approach or open-abdominal surgery because of avoiding intra-abdominal adhesions and assuring minimal surgical incision and postoperative course. The operation is conducted under left renal surgical position and modest pneumoperitoneum pressure. Laparoscopic approach is converted to open-abdominal surgery when the circulation deteriorates, or surgical field is interfered by bleeding. She was scheduled for retroperitoneal laparoscopic tumor excision.

General anesthesia was induced with fentanyl 8 µg/kg, midazolam 0.11 mg/kg, and rocuronium 1.1 mg/kg. We administered dobutamine 2 µg/kg/min, noradrenaline 0.03–0.12 µg/kg/min, vasopressin 0.5 units/h, and inhalation of NO 10 ppm to maintain hemodynamics after induction of anesthesia. We maintained CVP at approximately 20 mmHg. Anesthesia was maintained with sevoflurane 0.6–0.8 MAC and remifentanil 0.1–0.3 µg/kg/min. We adjusted PCV with a low PEEP of 5 mmHg to maintain a PaCO_2_ of approximately 35 mmHg. The operation was begun in the left renal position. Her hemodynamics were stable after CO_2_ insufflation for the retroperitoneal laparoscopic procedure, in which the pneumoperitoneum pressure was set at 6–8 mmHg. Although hemodynamic fluctuations occurred during tumor manipulation (approximately ± 10–20% of the mean BP compared to before manipulation), the patient was successfully managed with four bolus infusions of phenylephrine 0.1 mg. The operation was performed as scheduled.

## Discussion

In a Fontan circulation, the pulmonary blood flow is passively determined by a combination of CVP, left atrial pressure, systemic ventricular end-diastolic pressure, and PVR because of the lack of a responsible ventricle for determining pulmonary circulation. In such cases, PVR and intravascular volume must be controlled to maintain pulmonary circulation because Fontan physiology is preload dependent and requires low PVR [8, 13]. In particular, CVP and PVR may be the most important parameters in the perioperative management of Fontan circulation [8, 13–15]. We managed intraoperative CVP at preoperative levels under fluid infusion as previous case reports [13, 15] and utilizing NO to decrease PVR in all cases. In addition, we administered noradrenaline or vasopressin to maintain SVR when BP deteriorated even though CVP was maintained. In case 3, we administered dobutamine as BP was not maintained even though the noradrenaline and vasopressin. While we did not administer milrinone to avoid decreasing SVR and increasing HR for a failed Fontan circulation. Bigelow A. M. et al. reported that vasopressin administration was associated with a reduced transpulmonary gradient [16]. Administration of high-dose noradrenaline or phenylephrine might increase PVR [17]. Although high-dose vasopressin also has risk of mesenteric ischemia [18], vasopressin might be a useful vasopressor, and we could manage BP without increasing CVP in cases 2 and 3.

P-PGL are rare neuroendocrine tumors secreting high levels of catecholamines. Hypertensive crises, cardiac arrhythmias, and pulmonary edema are major adverse cardiovascular events that can potentially occur during surgery. Preoperative administration of an *α*-adrenergic receptor blocker is essential for preventing these adverse events [19]. Suffredini G. et al. and Lee H. C. et al. described methods of controlling hemodynamics during catecholamine surges; catecholamines secreted by P-PGL could cause massive increases in SVR followed by decreased cardiac output [8, 13]. Calcium blockers or *β*-blockers might be useful, as seen in our cases 1 and 2. Moreover, *α*-blockers also might be useful to manage hemodynamics during catecholamine surges [20].

To the best of our knowledge, this is the first case report of retroperitoneal laparoscopic resection for pheochromocytoma with failing Fontan circulation as case 3. Although the retroperitoneal approach only allows a relatively small operating space with nuclear anatomical landmarks and needs technical skill and experience, its use can help avoid intestinal interference and postoperative intestinal obstruction [21]. In particular, this approach is conducive to completing the operation without interference from intra-abdominal adhesions, as seen in case 3. Laparoscopic surgery is challenging for Fontan physiology because the pneumoperitoneum increases SVR and decreases venous return and cardiac output [8, 12, 14]. Although the pneumoperitoneum pressure was set at 6–8 mmHg, CVP and ABP were stable in our case. Perioperative discussions involving anesthesiologists, cardiologists, surgeons, and endocrinologists are essential in such cases.

## Supplementary Information

**Additional file 1.** Case reports describing anesthetic management of patients with Fontan physiology pheochromocytoma and paraganglioma resection.

## Data Availability

Not applicable.

## Abbreviations

ABP: Arterial blood pressure
APTT: Activated partial thromboplastin time
ASD: Atrial septal defect
AV: Atrioventricular
AVSD: Atrioventricular septal defect
BP: Blood pressure
ccTGA: Congenitally corrected transposition of great arteries
CI: Cardiac index
CVP: Central venous pressure
DORV: Double outlet right ventricle
dTGA: Dextro-transposition of great arteries
FFP: Fresh-frozen plasma
HLHS: Hypoplastic left ventricle syndrome
HR: Heart rate
ICU: Intensive care unit
IVPCA: Intravenous patient-controlled analgesia
MAC: Minimum alveolar concentration
MIBG: Metaiodobenzylguanidine
NA: Not available
NO: Nitric oxide
NR: Normal range
PA: Pulmonary atresia
PaCO_2_: Partial pressure of arterial carbon dioxide
PCV: Pressure control ventilation
PEEP: Positive end expiratory pressure
P-PGL: Pheochromocytoma and paraganglioma
PS: Pulmonary stenosis
PT-INR: International normalized ratio of partial thrombin time
PVR: Pulmonary vascular resistance
RCC: Red cell concentrate
SpO_2_: Oxygen saturation
SVEF: Systemic ventricular ejection fraction
SVR: Systemic vascular resistance
TA: Tricuspid atresia
TAPVC: Total anomalous pulmonary venous connection
TEE: Transesophageal echocardiography
VSD: Ventricular septal defect
