# Supplementary material for: Anesthetic management of pheochromocytoma and paraganglioma for patients with Fontan circulation: a case series
Source: JA Clin Rep. 2023 Mar 10;9:13. doi: 10.1186/s40981-023-00605-z (PMC10006357; doi:10.1186/s40981-023-00605-z)
Supplement: Supplementary file 1 — Additional file 1. Case reports describing anesthetic management of patients with Fontan physiology pheochromocytoma and paraganglioma resection. [file 40981_2023_605_MOESM1_ESM.docx]

**Supplement:** Case reports describing anesthetic management of patients with Fontan physiology pheochromocytoma and paraganglioma resection

| **Case report** | **Age (years) /sex** | **Underlying disease** | **Anesthetic induction** | **Maintenance** | **Monitoring** | **Laparoscopic or open** | **Nitric oxide** | **Inotropes** | **Intraoperative CVP**  **(mmHg)** | **Operation duration**  **(min)** | **Amount of bleeding**  **(g)** |
| --- | --- | --- | --- | --- | --- | --- | --- | --- | --- | --- | --- |
| **Tjeuw, et al. [3]** | 11/F | dTGA,  TA, PS,  ASD | Fentanyl,  diazepam,  vecuronium | Fentanyl,  vecuronium | ABP,  CVP | Open | - | - | 12 | 150 | NA |
| **Sparks, et al. [4]** | 27/F | HLHS,  DORV,  PA | Etomidate,  sufentanil,  rocuronium | Sufentanil,  isoflurane | ABP | Open | - | Fenoldopam | NA | 180 | Minimal |
| **Yuki, et al.**  **[5]** | 24/M | Holmes heart | Etomidate,  fentanyl,  vecuronium | Fentanyl,  midazolam,  pancuronium,  isoflurane | ABP,  CVP,  TEE | Open | - | Fenoldopam,  dopamine,  phenylephrine,  noradrenaline | 20 | 240 | 500 |
| **Lattendresse, et al. [6]** | 11/M | Large VSD | Etomidate,  rocuronium,  remifentanil | Sevoflurane,  remifentanil | ABP,  CVP,  TEE | Open | - | Phenylephrine | 13–15 | 480 | 350 |
| **Cherqaoui, et al. [7]** | 13/M | TA, PS, dysplastic single AV valve | Hydroxyzine,  propofol,  pemifentanil,  atracurium | Sevoflurane,  remifentanil | ABP,  CVP | Open | - | Noradrenaline | NA | NA | NA |
| **Lee, et al. [8]** | 18/M | HLHS, cAVSD, ccTGA, TAPVC | Midazolam,  remifentanil,  etomidate,  eocuronium | Sevoflurane,  remifentanil | ABP,  CVP,  TEE | Laparoscopic; converted to open | - | Milrinone,  nitroprusside,  dopamine,  noradrenaline | 15 | 490 | NA |
| **Haile, et al. [9]** | 25/M | PA | Fentanyl,  lidocaine,  propofol,  vecuronium | Isoflurane,  remifentanil | NA | Open | - | Epoprostenol,  nitroprusside  milrinone | NA | NA | NA |
| **Cummings, et al. [10]** | 36/F | PA | Propofol,  lidocaine,  fentanyl,  rocuronium | Isoflurane,  propofol | ABP, CVP,  TEE | Robot | - | - | NA | NA | 15 |
| **Sherburne, et al. [11]** | 16/NA | Hypoplastic right heart | Midazolam,  propofol,  fentanyl,  lidocaine,  rocuronium | Sevoflurane,  fentanyl,  rocuronium | NA | NA | - | Milrinone,  nitroprusside,  noradrenaline,  adrenaline | NA | 180 | 100 |
| **Masato, et al. [12]** | 39/F | HLHS,  PS | Midazolam,  fentanyl,  rocuronium | Sevoflurane,  remifentanil | ABP,  CVP,  TEE | Laparoscopic | + | Milrinone,  carperitide,  nitroglycerin,  noradrenaline,  Dobutamine | 14–22 | 225 | 74 |
| **Suffredini, et al. [13]** | 36/M | TA | Ketamine,  midazolam,  fentanyl,  vecuronium | Isoflurane,  epidural infusion of bupivacaine | ABP,  CVP,  TEE | Open | - | Nitroprusside | His baseline | NA | Minimal |
| **Suffredini, et al. [13]** | 35/F | Hypoplastic right heart,  VSD,  PS | Ketamine,  midazolam,  fentanyl,  vecuronium | Isoflurane,  epidural infusion of bupivacaine | ABP,  CVP,  TEE | Open | - | Nitroprusside | Her baseline | NA | Minimal |

ABP: arterial blood pressure, ASD: atrial septal defect, AV: atrioventricular, cAVSD: complete atrioventricular septal defect, ccTGA: congenitally corrected transposition of great arteries, CVP: central venous pressure, DORV: double outlet right ventricle, dTGA: dextro-transposition of great arteries, HLHS: hypoplastic left ventricle syndrome, NA: not available, PA: pulmonary atresia, PS: pulmonary artery stenosis, TA: tricuspid atresia, TAPVC: total anomalous pulmonary venous connection, TEE: transesophageal echocardiography, VSD: ventricular septal defect
